# A Rare Case of New-Onset Ulcerative Colitis following Initiation of Secukinumab

**DOI:** 10.1155/2019/2975631

**Published:** 2019-07-31

**Authors:** Ted George Achufusi, Prateek S. Harnee, Sekou Rawlins

**Affiliations:** ^1^Department of Internal Medicine, SUNY Upstate Medical University, 750 East Adams Street, Syracuse, NY 13210, USA; ^2^Department of Gastroenterology, SUNY Upstate Medical University, 750 East Adams Street, Syracuse, NY 13210, USA

## Abstract

Secukinumab is an IgG monoclonal antibody widely used for treatment of ankylosing spondylitis, psoriasis, and psoriatic arthritis. Recently, there has been increasing controversy regarding potential adverse effects of the drug especially in those with underlying inflammatory bowel disease. We present the case of a young male patient who developed severe new-onset ulcerative colitis following initiation of secukinumab for psoriasis, with excellent response and rapid resolution of symptoms with infliximab.

## 1. Introduction

The pathogenesis of IBD involves a group of proinflammatory cytokines, one of them being IL-17 [1]. Secukinumab is an IgG monoclonal antibody directed against interleukin 17-A approved for treatment of psoriasis, psoriatic arthritis, and ankylosing spondylitis [2, 3]. Despite its immunomodulating properties, data suggest that secukinumab has no role in treating any form of inflammatory bowel disease; however, the debate over potential exacerbation of underlying IBD and new-onset fulminant IBD following secukinumab therapy is still ongoing [1, 3–6].

## 2. Case

A 39-year-old man with a past medical history of psoriasis treated with secukinumab by his dermatologist presented from an outside hospital for further evaluation of abdominal pain and bloody diarrhea. The patient initially developed suprapubic abdominal pain with associated hematochezia and tenesmus six months after beginning secukinumab treatment. The patient had a history of severe plaque psoriasis with initial onset about 5 years ago. He did not respond to initial therapies and hence was started on secukinumab which induced remission.

After developing abdominal pain and fevers, he was referred to a gastroenterologist for additional evaluation especially given close to 30 lbs of weight loss in one month. Abdominal CT scan was significant for a few diverticula, but there was no sign of active inflammation. However, on initial colonoscopy, there was mild inflammation of the sigmoid colon. He was started on ciprofloxacin and metronidazole for suspected infectious colitis with no relief in his symptoms.

**Figure 1:**
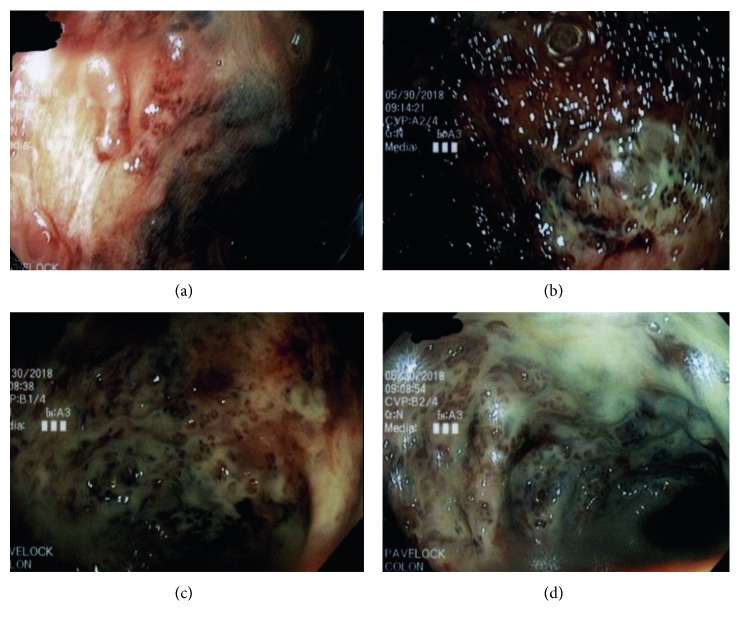
Endoscopic evaluation demonstrating severe colitis throughout the colon.

**Figure 2:**
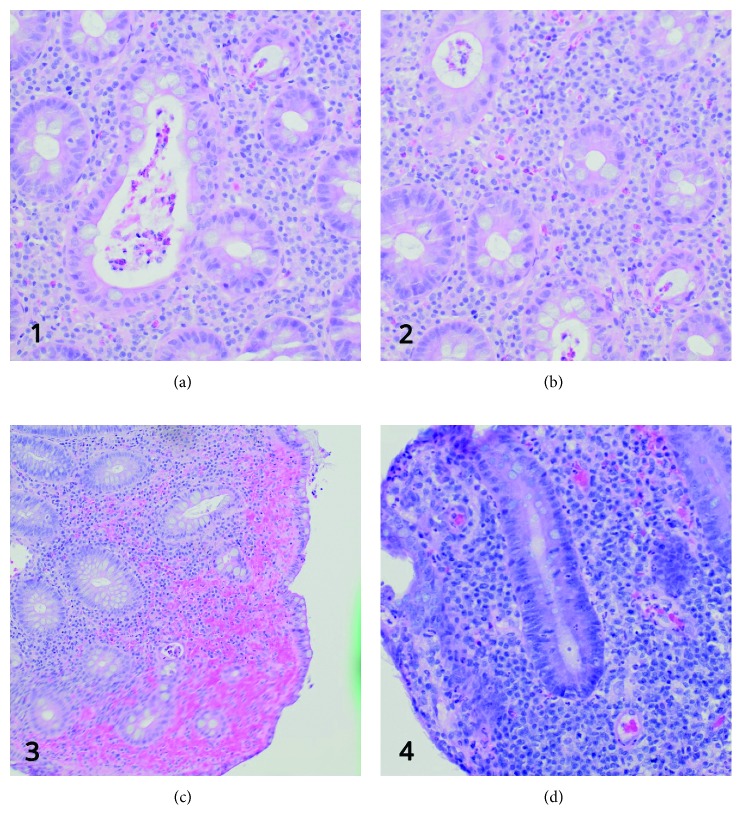
Histological features. (a, b) Distortion of the crypt architecture, inflammation of crypts (cryptitis), frank crypt abscesses, and hemorrhage or inflammatory cells in the lamina propria. (c) Intense inflammation of the mucosa is seen. (d) The colonic mucosal epithelium demonstrates loss of goblet cells. The shape of the crypts is distorted. Also, there are features of early dysplasia towards the bottom of the crypts as seen by the deep staining and crowded nuclei.

Five days later, the patient presented to his local emergency department complaining of worsening abdominal pain, fever, chills, and multiple loose bloody bowel movements per day. Repeat CT abdomen showed thickening of the descending and sigmoid colon. He was again started on an antibiotic treatment with piperacillin-tazobactam along with steroids for possible autoimmune colitis. Flexible sigmoidoscopy showed ulceration of the splenic flexure, moderate to severe active colitis, ulceration at 30 cm, and active colitis in the rectum (Figure 1). Grossly, there was no inflammation past the transverse colon. Biopsies showed a collection of neutrophils within crypt lumens and architectural distortion, including shortening of crypts and variation in the sizes and shapes of crypts (Figure 2). The patient remained in the hospital for an additional 10 days with no significant improvement in his clinical condition and was transferred to our institution for further evaluation.

On presentation, labs were significant for a white count of 17000 per microliter with bandemia and an elevated CRP of 40 mg/dl. He continued to have 5-6 blood-tinged stools a day, and his abdominal pain did not improve despite being on the steroids. Subsequently, he was started on infliximab to facilitate symptom relief, remission without steroids, and intestinal healing.

There was a dramatic improvement in his clinical symptoms with respect to abdominal pain, frequency of diarrhea, and amount of blood in his stools by the time of discharge. Follow-up colonoscopy two weeks later showed significant improvement as there were no deep ulcerations, which were grossly observed on prior colonoscopy. Biopsies taken from the sigmoid and rectal mucosa showed the distorted crypt architecture as well as neutrophilic infiltration, but this was much less pronounced than that prior to therapy. Over the next few weeks, the patient had gradual improvement in bloody diarrhea to two times per day and reported a 10-pound weight gain within a four-week period since initiation of infliximab, which was continued by his gastroenterologist following discharge from our hospital. After halting secukinumab, his psoriasis has been well controlled on apremilast, commonly known as Otezla, which belongs to the antirheumatic drug class.

## 3. Discussion

The amount of literature describing the correlation between the slow and the rapid onset of IBD following secukinumab treatment is very limited [4, 5]. The pathophysiology behind IBD involves upregulation of proinflammatory and immune-regulatory cytokines within the mucosa of the small and large intestines. One of the cytokines thought to contribute to the development of IBD is IL-17, which has strong proinflammatory activity [7–10]. Expression of IL-17 is not seen in patients with healthy colonic mucosa or patients suffering from infectious or ischemic colitis [8]. However, the expression of IL-17 mRNA has been shown to be significantly increased in patients with active ulcerative colitis and Crohn's disease [8, 11, 12]. Given this finding, it was initially perceived secukinumab would have therapeutic potential for treating IBD [1, 13]. However, there have been reports of increased adverse effects among IBD patients who are undergoing treatment with secukinumab for other conditions [4, 5]. Interestingly, this was also demonstrated with brodalumab, another monoclonal antibody drug against the IL-17 receptor that has been shown to have adverse effects on patients with Crohn's disease [14]. Similarly, another clinical study which tested the efficacy of secukinumab in treatment of Crohn's disease demonstrated that blocking IL-17A in Crohn's disease patients exacerbated their symptoms and was associated with increased inflammatory markers over baseline [15]. Despite the existence of literature documenting these cases, many still believe that there is absolutely no risk of precipitating IBD with secukinumab use [16]. In the phase 3 trial for secukinumab, of the 3430 patients exposed to the drug over the entire treatment period for up to 52 weeks (2725 patient-years), there were 3 cases (0.11 per 100 patient-years) of exacerbation of Crohn's disease, 2 cases (0.08 per 100 patient-years) of exacerbation of ulcerative colitis, and 2 cases (0.08 per 100 patient-years) of new-onset ulcerative colitis [17]. A 2017 review which looked at several studies in which patients were treated with secukinumab for ankylosing spondylitis, psoriasis, or psoriatic arthritis concluded that there was no increased risk of developing IBD with secukinumab use [16].

More research needs to be done to truly understand the exact pathogenesis of ulcerative colitis following treatment with secukinumab. Currently, we know that it selectively binds to IL-17A, inhibiting interaction with the IL-17 receptor and thus the release of proinflammatory cytokines and chemokines [18, 19]. However, it is difficult to predict the effect of cytokine blockade on IBD patients because of the unpredictability of the immune response. Previous studies done on mice demonstrated that blocking IL-17 causes worsening of colitis by increasing tumor necrosis factor-alpha, interferon-gamma, and IL-6, proteins and cytokines that promote the inflammatory response [20]. This mechanism might have been in play when it came to the development of symptoms in our patient. However, it is also important to note that, despite its proinflammatory activity, IL-17 might also have a beneficial effect within the gut. In animal models, IL-17A promoted epithelial barrier function by regulating tight junction proteins such as occludin, protecting mice from excessive gut permeability after epithelial injury [21]. This points towards a delicate balance between a potentially favorable effect and a detrimental effect of IL-17 within the gut.

One of the challenges with treating this patient was that secukinumab has an extremely long half-life estimated at around 24–31 days [18–20, 22]. This might explain why the patient had ongoing inflammation, albeit improved, at his follow-up colonoscopy after he was discharged from our care. The patient was most likely at the end of the half-life of secukinumab, based on the history he provided us. It is important for clinicians to be cautious when treating patients with infliximab, especially if the patient was already on another immunosuppressant prior to starting therapy. Our patient received his first infliximab injection within a month of his last dose of secukinumab. This can cause excessive immunosuppression and put patients at risk for infection; therefore, it is vital to screen for viral immunity and test for tuberculosis prior to initiating treatment. Interestingly, literature has emerged discussing the potential of combining biologic therapies in patients with IBD and other immune-mediated inflammatory diseases. In refractory cases, vedolizumab was successfully used in association with infliximab for treatment of Crohn's disease patients with other concomitant autoimmune diseases. These data suggest that combination biologic therapy might represent a therapeutic option in selected patients with IBD; however, larger studies are needed [23].

It is also important to consider alternative diagnoses such as ischemic and infectious colitis as both can present with similar symptoms. Initially, there was suspicion for ischemia based on gross appearance on colonoscopy; however, given a normal CT angiography, this was unlikely. Additionally, given the fact that he had been on immunosuppressive therapy for psoriasis, it did put him at increased risk for infection. Testing for CMV colitis was negative, and no inclusion bodies were seen on biopsy. All other infectious workups were negative including *C. difficile*, and multiple cultures of his stool were taken all of which came back negative. It is important to remember that repeat imaging is unusual; however, given that he presented to his local emergency department, it is not surprising that he underwent repeat imaging as his prior CT scan was taken at an outside institution. The gold standard for diagnosing ulcerative colitis remains colonoscopy with architectural changes on biopsy.

Another important consideration regarding the patient's history is that he was completely asymptomatic prior to starting secukinumab treatment, and therefore, this appears to be a de novo case of UC rather than an exacerbation of existing UC. This would be an extremely rare finding as most previously documented cases describe exacerbation of previously diagnosed IBD following secukinumab treatment. However, there is no definitive way to say whether this patient had underlying UC prior to starting therapy. There is a small subset of patients with UC who live symptom-free and are not diagnosed until routine screening colonoscopies. There is a chance our patient was suffering from mild UC that caused him to be asymptomatic and that secukinumab exacerbated his condition. Furthermore, our patient could have developed IBD even without starting secukinumab, considering that he was asymptomatic for months following initiation of the drug. However, considering no positive family history and rapid resolution of his symptoms after halting the medication, we cannot exclude the possibility that his symptoms were solely due to secukinumab use.

This case presents an important challenge for clinicians who are treating their patients with secukinumab and other IL-17 antagonists. Given the potential risk of IBD exacerbation, clinicians should consider evaluating patients for underlying inflammatory bowel disease prior to starting any IL-17 antagonist and close monitoring of those who are already being treated with these agents. With the high number of targeted therapies introduced to the market for various inflammatory conditions, it is vital for clinicians to be aware of the limitations and potential side effects of these agents and whether their use is appropriate for those with underlying inflammatory bowel disease.
