# Corrigendum to “A Rare Case of New-Onset Ulcerative Colitis following Initiation of Secukinumab”

**DOI:** 10.1155/2019/3689298

**Published:** 2019-11-20

**Authors:** Ted George Achufusi, Prateek S. Harne, Sekou Rawlins

**Affiliations:** ^1^Department of Internal Medicine, SUNY Upstate Medical University, 750 East Adams Street, Syracuse, NY 13210, USA; ^2^Department of Gastroenterology, SUNY Upstate Medical University, 750 East Adams Street, Syracuse, NY 13210, USA

In the article titled “A Rare Case of New-Onset Ulcerative Colitis following Initiation of Secukinumab” [1], the name of the second author was given incorrectly as Prateek S. Harnee. The author's name should have been written as Prateek S. Harne. The revised authors' list is shown above.
